# Assessing the effect of an evidence-based patient online educational tool for people with multiple sclerosis called UMIMS—understanding magnetic resonance imaging in multiple sclerosis: study protocol for a double-blind, randomized controlled trial

**DOI:** 10.1186/s13063-020-04855-5

**Published:** 2020-12-09

**Authors:** Insa Schiffmann, Magalie Freund, Eik Vettorazzi, Jan-Patrick Stellmann, Susanne Heyer-Borchelt, Marie D’Hooghe, Vivien Häußler, Anne Christin Rahn, Christoph Heesen

**Affiliations:** 1 Institute of Neuroimmunology and Multiple Sclerosis (INIMS), University Medical Centre Hamburg-Eppendorf (UKE), Hamburg, Germany; 2 Department of Neurology, University Medical Centre Hamburg-Eppendorf (UKE), Hamburg, Germany; 3 Department of Medical Biometry and Epidemiology, University Medical Centre Hamburg-Eppendorf, Hamburg, Germany; 4 APHM, Hopital de la Timone, CEMEREM, Marseille, France; 5 Aix Marseille Université, CNRS, CRMBM, UMR 7339, Marseille, France; 6 Centre for Neurosciences, Vrije Universiteit Brussel, Brussels, Belgium; 7 National MS Centre Melsbroek, Melsbroek, Belgium; 8 Department of Health Services Research, Carl von Ossietzky University Oldenburg, Oldenburg, Germany

**Keywords:** Multiple sclerosis, Evidence-based patient information, Magnetic resonance imaging, Risk knowledge, Disease-specific knowledge, Autonomy preferences, Shared decision-making, Randomized controlled trial, Process evaluation

## Abstract

**Background:**

While magnetic resonance imaging (MRI) plays a major role in the lives of people with multiple sclerosis (pwMS), studies have shown that MRI-specific knowledge in pwMS is limited. Moreover, poor knowledge was associated with negative feelings towards MRI (e.g. anxiety concerning MRI scan). Because information sources about MRI in MS for pwMS are not available, we designed and evaluated an evidence-based online educational platform about MRI in MS called “Understanding MRI in MS” (UMIMS). Based on a pilot study in *n*= 79 subjects, an educational intervention was found to be feasible and effective. We hypothesize that MRI-specific knowledge can be increased by using UMIMS and that, subsequently, negative feelings towards MRI will be reduced and shared decision-making competences increased.

**Methods:**

This randomized, controlled, double-blinded trial (RCT) will recruit *n* = 120 pwMS. The intervention group will receive access to UMIMS. The control group will get access to a specifically developed control website, which visually imitates UMIMS and contains the standard information available by several MS self-help organizations. The change in MRI-specific knowledge assessed via the MRI-risk knowledge questionnaire (MRI-RIKNO) after the intervention is the primary endpoint at 2 weeks. Several secondary endpoints will be assessed at different timepoints throughout the study, e.g. emotions towards MRI, autonomy preferences, threat by MS and shared decision-making competences. The study includes a process evaluation.

**Discussion:**

The aim of this RCT is to prove that access to an education tool on MRI in MS will increase pwMS’ MRI-specific knowledge and enhance shared decision-making when discussing decisions involving MRI results during patient-physician encounters.

**Trial registration:**

Clinicaltrials.gov NCT03872583. Registered on 13 March 2019.

## Background

Multiple sclerosis (MS) is a chronic demyelinating disease mainly affecting young adults; in Germany, more than 200,000 people are afflicted by MS [1]. Magnetic resonance imaging (MRI) plays a major role in the diagnosis [2], predicting the prognosis [3] and controlling treatment effectiveness during the course of the disease [4]. Especially at the beginning of the disease, pwMS may have several MRIs within 12 months, and even in the absence of clinical disease activity, annual MRI monitoring has been suggested (Magnetic Resonance Imaging in MS (MAGNIMS)-Network) [5]. While the number and location of MS lesions in MRI after the first clinical event has a limited predictive value for the progression in the following 7 or 20 years [6], lesion load during the course of the disease is virtually unrelated to clinical appearance of a patient. This discrepancy is called the “clinico-radiological paradox” [7]. There is no international consensus about handling the occurrence of new MS lesions when assessing treatment effectiveness. For example, while there is evidence that the appearance of more than 3 new T2 lesions predicts treatment failure in pwMS treated with beta-interferons [4], many neurologists follow the “no evidence of disease activity” (NEDA) concept and may change treatment even after the appearance of a single new silent T2 lesion [8].

Therefore, oftentimes, when MRI results are discussed in the context of initiation or change of a disease-modifying drug (DMD), there is no clear medically superior option. These so-called preference-sensitive decisions are predestined for a shared decision-making (SDM) approach, in which physician and patient discuss options together. A prerequisite for SDM is a sufficient disease-specific knowledge. In pwMS, however, this knowledge has proven to be only moderate with around 60% of correctly answered questions in MS-specific [9, 10] and MRI-specific knowledge questionnaires [11]. Apart from being a necessity for medical decision-making, a high disease-specific knowledge can also influence patients’ emotions: For example, a low disease-specific knowledge was associated with greater levels of fear in patients with chronic obstructive pulmonary disease [12], and patient education has also been shown to decrease anxiety before medical procedures, e.g. in the perioperative setting [13]. Therefore, increasing MRI knowledge in pwMS might not only lead to a better understanding of their disease and increase shared decision-making, it may also decrease anxiety concerning MRI.

To our knowledge, evidence-based and patient-friendly information on MRI in MS is currently not available, hindering patient participation. We have therefore developed an online patient education tool on MRI in MS called “Understanding MRI in MS” (UMIMS). This randomized controlled trial (RCT) aims to assess the effect of this online education tool on knowledge about and emotions towards MRI in *n* = 120 pwMS.

### Aims and objective

We hypothesize that access to an online education tool about MRI in MS will lead to an increase in MRI-specific knowledge in pwMS. Increased knowledge will be accompanied by a decrease of fear of MRI and increased feeling of competence during patient-physician encounters. The intervention will enhance patient empowerment and increase patients’ desire for participation in MRI-related decisions. Finally, it will increase SDM when considering MRI-activity-based treatment changes or decisions about subsequent MRIs during clinical interactions. At large, this study is guided by the principles of evidence-based medicine (EBM) [14] and evidence-based patient information (EBPI) [15] and the Medical Research Council (MRC) framework for developing and evaluating complex interventions [16].

## Methods

### Design

The UMIMS trial will be carried out as a double-blind, superiority randomized controlled trial. Following the MRC guidelines for the development and evaluation of complex interventions [16], the website and the outcome measures used in the RCT were pre-tested in a feasibility study with *n* = 79 participants recruited via the website of the German MS society. In detail, the feasibility study tested if patients had technical issues accessing or using the website, asked how much time patients spent on it and if patients found the content understandable, interesting and relevant. Furthermore, the main study will be accompanied by a process evaluation [16].

### Study setting

To ensure a more diverse patient population, the study will be conducted in 5 German clinics specialized in MS care (3 university clinics, and 2 private practices) throughout Germany.

### Eligibility criteria

PwMS are eligible to participate if they are older than 18 years and have been diagnosed with a relapsing-remitting MS (RRMS) according to the McDonald criteria [2] within the previous 10 years and have an active disease course (i.e. treatment change or new T2 lesion within the previous year) *or* a clinically isolated syndrome (CIS) with at least one MS-typical T2 lesion.

In order to realistically assess whether the education tool changes the attitude towards MRI, patients can only be included if they are scheduled to receive an MRI within 2 weeks to 6 months following randomization.

Because the study will use the internet for information provision and data collection, only patients with access to the internet will be included.

### Patient exclusion criteria

We assume that MRI results have the greatest impact on people with active RRMS or suspected MS (e.g. resulting in treatment initiation or confirmation of diagnosis) and therefore decided to exclude patients with (even active) secondary-progressive or primary-progressive MS from participation. Additionally, severe cognitive deficit or major psychiatric illness affecting information uptake, any suspected central nervous system disease other than MS, contraindications for MRI and pregnancy are reasons for exclusion.

### Interventions

#### Intervention group (IG)

After answering the baseline questionnaires and randomization, patients in the intervention group will receive access to the newly developed, evidence-based, online education tool “Understanding MRI in MS” (UMIMS). The tool was developed based on previous research [11] and the input of pwMS, MRI experts and expert patients. It consists of 3 sections:

1. “About MRI” (MRI education)
2. “Learning to read” (interactive MRI training with real MRI images)
3. Training (MRI quiz)

“About MRI” covers topics, which emerged during the interviews with pwMS and/or were deemed relevant by the expert patients, MRI experts or the research team. Expert patients are defined as people affected by an illness, that, among other characteristics, have both personal and experiental experiences with the disease, are knowledgeable in symptoms and treatment of it and have active roles or even hold responsibilities in self-help organizations (e.g. as board members) [17]. It includes evidence-based information on the importance of MRI for prognosis (e.g. impact of number and location of MS lesions), diagnosis (e.g. explanation of the McDonald criteria) and treatment control in MS (i.e. definition of non-responders), the MRI procedure (e.g. duration and scanning procedure), contrast agents (i.e. use and risks of gadolinium), basic neuroanatomy, lesion knowledge (i.e. configuration and types of MS lesions) and MRI sequences. Easy-to-understand figures, videos and explanations of technical terms simplify the learning process. All evidence sources are cited. In “Learning to read”, users are presented with 3 different doctor’s letters, which are translated into layman’s terms and 7 original MRI images with a step-by-step explanation for interpretation of the results. In the “Training” section, patients can test their acquired knowledge in a quiz.

### Control group (CG)

After answering the baseline questionnaires and randomization, patients in the control group will receive access to a newly created website providing standard information on MRI in the same design as the UMIMS website. The standard information consists of the content that was freely available on the websites of several European as well as major English-speaking MS self-help organizations (Germany, France, Belgium, Netherlands, Great Britain, Canada, Australia, USA; time of access: September 2018). All topics that were permanently hosted on the websites were included; single articles, which were only available via a separate search were not included. The topics are as follows: importance of MRI for diagnosis (i.e. short explanation of the McDonald criteria) and treatment control in MS (i.e. that MR images are used for decision-making after treatment initiation), MRI procedure (i.e. duration, contraindications for MRI scans and basic procedure of the scan) and contrast agents (i.e. what contrast agents are used for).

### Outcomes

For a list of the major endpoints of the UMIMS trial, see Table 1.

**Table 1:** Major endpoints

Instrument	Measurement time point				
	Enrollment	Allocation	Post-allocation		
	*t*_−1_	*t*_0_	*t*_1_	*t*_2_	*t*_3_
Eligibility screen	x				s
Informed consent	x				
Allocation	x				
Sociodemographic data	x	x			
PDDS		x			
MS-related data and resource use		x			
MRI-RIKNO		x^a^	x		
Subjective knowledge		x	x		
MRI-EMA		x	x	x	
CPS		x	x	x	
Numeracy		x			
Threat by MS		x	x		
HADS		x	x		
Process evaluation			x	x (patient and physician)	x
MAPPIN’SDM				x (patient and physician)	
MAPPIN’SDM audio (*n* = 5 CG/IG)				x (external rater)	
Treatment/MRI decision				x	x

*t*_*1*_ 2 weeks after allocation and access to the intervention/control website, *t*_*2*_ immediately after patient-physician encounter, *t*_*3*_ 6 months after patient-physician encounter, *CG* control group, *CPS* Control Preference Scale, *HADS* Hospital Anxiety and Depression Scale, *IG* intervention group, *MAPPIN’SDM* Multifocal Approach to Sharing in Shared Decision Making, *MRI-EMA* magnetic resonance imaging-emotions and attitude questionnaire, *MRI-RIKNO* magnetic resonance imaging-risk knowledge questionnaire, *PDDS* patient-determined disease steps

^a^Primary endpoint

#### Primary endpoint

The primary endpoint is MRI-risk knowledge measured by the MRI-risk knowledge questionnaire 2.0 (MRI-RIKNO) [11]. It comprises *n* = 14 mainly multiple-choice items (maximum score of *n* = 22) concerning the meaning of MRI for diagnosis, prognosis and treatment control. It aims to assess pwMS’ basic understanding (e.g. basic neuroanatomy, recognizing lesions on MR images, procedure of the scan) as well as knowledge on relevant features of MRI results in MS (e.g. meaning of new lesions in an MRI for prognosis or use of contrast agents). MRI-risk knowledge will be assessed twice during the trial: *t*_0_ (allocation) and *t*_1_ (after a 2-week access to the intervention or control website). The primary endpoint is change of MRI-RIKNO score from baseline to *t*_1_. The study is powered to detect a 10% difference in the proportion of correct answers.

#### Secondary endpoints

Emotions and attitude towards MRI will be assessed using the validated questionnaire “MRI-emotions and attitude” (MRI-EMA) at *t*_0_, *t*_1_ and *t*_2_ [18]. Autonomy preferences will be assessed using the Control Preference Scale (CPS) [19] (moderate internal consistency and good convergent validity [20]) before and after the (control) intervention (at *t*_0_ and *t*_1_) as well as after the patient-physician encounter (*t*_2_). Perceived involvement in decisional encounters concerning MRI results and their consequences will be evaluated with the Multifocal Approach to Sharing in Shared Decision Making (MAPPIN’SDM; inter-rater-reliabilities in the observer scales and internal consistencies high to excellent) evaluation [21]; applying a newly developed short version. For a subgroup of *n* = 5 participants of each group, who have specifically consented to it, the encounter will be audiotaped and the degree of SDM will be assessed via an external rater using the MAPPIN’SDM. Decisions on future MRIs and treatment changes as well as acceptance of the intervention will be assessed from patients using a standardized questionnaire immediately (*t*_2_) and 6 months after the patient-physician encounter (*t*_3_) (for both the intervention group (IG) and control group (CG)). Quality of life (QoL) will be assessed using the subscales fatigue, cognition, visual impairment, communication and mood of the HAmburg QUAlity of life questionnaire in MS (HAQUAMS) [22] (internal consistency and retest coefficients high).

#### Tertiary outcomes (control and safety parameters)

Anxiety and depression will be assessed as a control parameter using the Hospital Anxiety and Depression Scale (HADS) [23] will be assessed as a control parameter. Occurrence of relapses will be evaluated at baseline (*t*_0_), after patient-physician encounter (*t*_2_) and at the 6-month follow-up (*t*_3_) (for both the IG and CG) using a standardized questionnaire.

### Participant timeline

**Fig. 1:**
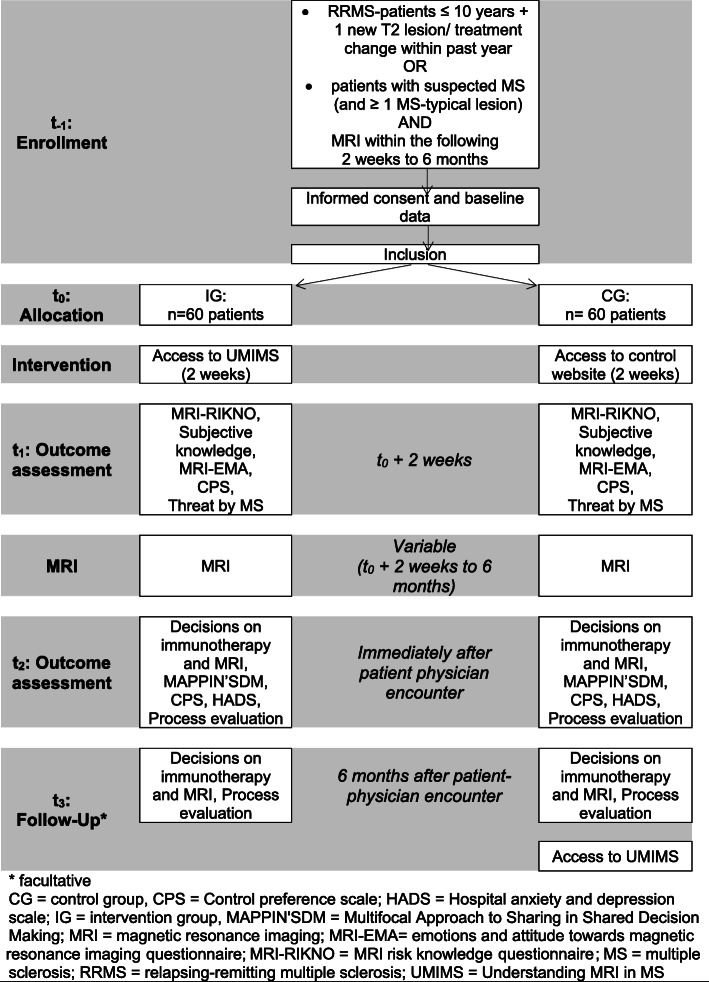
RCT design

For a presentation of the flow of the UMIMS trial, see Fig. 1.

#### Baseline data

After inclusion and random allocation to either the IG or CG, all patients will answer baseline questionnaires that assess demographic and clinical data, MRI knowledge (using the MRI-RIKNO), emotions and attitude towards MRI (using the MRI-EMA), autonomy preferences (using the CPS), numeracy and general internet use. Patients in the IG will receive an access code to the education tool UMIMS, and patients in the CG will receive a similar access code to the control website. The study nurse or other medical personnel will schedule the MRI appointment and the subsequent patient-physician encounter.

#### Post-MRI patient-physician encounter

Next, patients receive a 2-week access to either UMIMS or the control website; at the end of this period, the primary and several secondary endpoints are assessed. The MRI takes place in a 2-week to 6-month window after randomization. Participants then return to the study centre to discuss their MRI results. SDM concerning decisions based on MRI findings will be assessed via the MAPPIN’SDM questionnaire [21] and the actual decisions will be noted. All participants will be asked whether they agree to be audiotaped during the patient-physician encounter. The aim is to externally evaluate the SDM process in a subgroup of at least *n* = 5 patients from both groups. Both, physicians and patients, will answer questions for the process evaluation.

#### Follow-up

Six months after the patient-physician encounter, participants will be called to assess if the MRI-based decisions have led to any behavioural action, i.e. a new MRI or treatment change. Patients will be asked for their assumed group allocation. Unblinding might occur, if participants suspect to be in the control group due to the limited amount of information on the control website.

### Sample size

The primary endpoint of the UMIMS trial is the difference of change in MRI-specific risk knowledge between baseline and *t*_1_ between IG and CG. The sample size calculation is based on data from prior studies comparing *n* = 120 pwMS with and *n* = 497 pwMS without access to the educational program [18]. PwMS with access to the education program answered 75% of the MRI-RIKNO questionnaire correctly (16.3 out of 22 possible points, standard deviation (SD) ± 3.0), while participants without access answered 65% of questions correctly (14.5 of 22 possible points, SD ± 3.0). In order to detect this difference with a power of 90% and a significance level of alpha = 0.05, *n* = 49 patients in each group will be needed. Assuming a dropout rate of 20%, *n* = 11 additional participants will be needed, accounting for a total of *n* = 120 participants. In most of our previous trials on EBPIs, loss to follow-up was less than 10%. Therefore, 20% seems a realistic and conservative assumption.

### Recruitment, screening, allocation and blinding

#### Recruitment

Consecutive patients will be recruited by treating physicians in the 5 participating centres.

#### Screening

To minimize selection bias during the enrollment period, physicians are asked to consider every patient they see in a patient-physician encounter for the study. Eligible patients will be invited to participate and receive information about the study. Informed consent will be obtained by treating physicians from patients fulfilling the inclusion criteria after they have had enough time to read the study information and ask questions; participants then receive a pseudonym. Suitable patients, who are not willing to participate in the study, will be asked for the reason.

#### Allocation

Permuted-block randomization will be computer generated and performed by a statistician not involved in the conduct of the study; the randomization list is stored in a separate location not accessible to personnel responsible for the trial, and recruitment of new patients for randomization will be communicated via telephone. In a previous study, analysis of covariance did not reveal an influence of sociodemographic variables on MRI knowledge; therefore, no stratification will take place except for study site. Study materials, including the randomly assigned logins to the intervention or control website, will be provided by a member of the research team that is not involved in any outcome assessment or the analysis of the study.

#### Blinding

In the consent form, patients will be informed, that 2 different types of information will be tested. Blinding is achieved by providing a control group website featuring standard information on MRI in MS but following the same graphical format as the active intervention. Physicians and study nurses at the recruiting centres as well as 2 external raters for SDM during the patient-physician encounter will be blinded.

### Relevant concomitant care

#### Physician encounters

Patients are free to consult a physician and receive treatment for e.g. relapses at any point of the study. Any physician encounter will be documented.

#### Technical support

An employee that is not involved in the analysis of the study will answer any e-mails from participants reporting e.g. technical problems concerning the website. During this contact, content of the website (e.g. if a participant does not understand a certain figure and asks for an explanation) will not be discussed.

### Criteria for discontinuation

#### Adverse events

The intervention website contains complex medical information, which has the potential to overwhelm participants. Additionally, it provides information on the prognostic value of MRI and participants may learn that they fulfil negative prognostic criteria. However, our previous work has shown that pwMS understand complex medical information and are able to cope with negative information [24]. We do not foresee any other harm of the intervention. As relevant adverse events are unlikely, a data monitoring committee does not exist, no interim analyses are planned and no stopping rules will be applied. Nevertheless, safety measures are applied as tertiary endpoints to control for anxiety and depression. There is no anticipated harm and compensation for trial participation and no systematic post-trial care.

#### Patient withdrawal and non-adherence

At any point, patients in both groups can quit the study. Patients who withdraw from the study will be asked whether they agree to continue to fill in a limited set of questionnaires related to the primary study outcome. The data of non-adherent participants (e.g. with missing questionnaires) will be included in the intention-to-treat analysis.

#### Strategies to improve adherence

If appropriate, patients will be asked to fill in a questionnaire in the outpatient clinic directly after an encounter. When questionnaires have to be answered independently of an appointment, patients will be contacted by e-mail by a member of the coordinating centre in Hamburg and asked to complete the questionnaires within a specified time period. Patients that miss the completion will again be reminded by e-mail and telephone. If patients miss the physician appointment to discuss their MRI results, they will be contacted by the study nurse to arrange a new appointment.

To ensure the use of the education website, a study nurse will call the participants in the control and intervention group shortly after inclusion and encourage them to use the education tool. Additionally, participants will receive biweekly e-mail reminders concerning the tool.

### Data collection methods

Data will be collected at 4 time points using paper-pencil questionnaires (see Table 1). Follow-up data will be collected by telephone using trained and blinded interviewers after 6 months. Paper-based data is stored securely and pseudonymized at the coordinating centre. Data will be digitalized and evaluated for plausibility and quality (e.g. double data entry) by two employees of the coordinating centre independent from the study. Data will then be stored on secured servers at the coordinating centre and access will be granted to members of the trial team and the statistician after completion of the trial. Additional data management procedures (e.g. storage duration) can be found in the study protocol submitted to the Hamburg Chamber of Physicians (approval number: PV5722) as well as the patient consent form.

### Study supervision

Day-to-day delivery and conduct of the trial is performed by the coordinating researcher and a study nurse of the coordinating centre (Trial Management Group (TMG)). Treating physicians at the respective participating centres are responsible for recruitment of participants and obtaining written consent. The principle investigator, coordinating researcher and a study nurse monitor and supervise the trial, meet every 3 months to discuss its progress and update relevant parties (i.e. sponsors, trial registry, participants), i.e. take over the responsibilities of the Trial Steering Committee (TSC). Due to the low-risk intervention, an independent TSC, Data Monitoring Committee (DMC) or meeting of a Stakeholder and Public Involvement Group (SPIG) were not deemed necessary.

### Statistical methods

Continuous data will be described using means and standard deviations (SDs) and compared using Student’s *t* test. Categorical data will be presented using contingency tables and raw percentages and will be compared using Fisher’s exact test.

The primary endpoint, change in MRI-RIKNO from *t*_0_ to *t*_1_, will be analysed using an ANCOVA model with adjustment for baseline. Secondary endpoints will be analysed accordingly depending on the scale of measurement either by ANCOVA models for continuous endpoints or (ordinal) logistic regression models for dichotomous or ordinal endpoints.

It is planned to perform subgroup analysis of the 2 groups of patients included in the trial: first, those with an RRMS diagnosis of less than 10 years and active disease, and second, those with suspected MS. We will report causes for study withdrawal for each patient to clarify whether there are any differences between the intervention and control groups.

All data will be analysed on an intention-to-treat as well as per-protocol basis. In addition, sensitivity analyses will be performed to evaluate the robustness of study results and to explore different imputation techniques. Altman [25] addressed that there is no ideal method to address missing data. Therefore, different common imputation techniques [26] will be applied and reported with as well as without imputation techniques as suggested by Altman [25]. Best- and worst-case scenarios for dichotomous outcomes and multiple imputation techniques will be conducted in the sensitivity analysis [27].

### Process evaluation

This RCT will be accompanied by a process evaluation to measure the intervention’s fidelity and determine reasons for an (in)effective study outcome, following the guidelines of the MRC [16, 28]. The process evaluation will be used to investigate study processes related to participants, physicians and the context and setting of the study. The framework of the process evaluation is based on a previous study including a process evaluation [29] as well as guidelines by Moore et al. [30].

In this process evaluation, quantitative elements will be used to explore expected and unexpected events arising from the intervention. The overall aim is to uncover barriers and facilitators for reaching study goals and to explore mechanisms that lead to explanations for failure or success of the intervention.

In detail, the objectives are to:

1. Explore the reaction of individuals (such as user-friendliness of, hours spent on and feelings evoked by the website)
2. Detect barriers and facilitators for the intervention’s delivery (such as technical problems with the website) and for the dose received by participants (such as disease-related or internet-related problems participants may experience)
3. Find barriers and facilitators of study termination, participation and retention
4. Analyse reasons why study elements work or do not work out as planned
5. Reveal contamination of the intervention and control groups (such as the use of other information materials other than those provided during the trial)
6. Identify unintended consequences of the study (such as depression and anxiety)

All questionnaires have been constructed by the research team and were tailored specially to the study. The content of this process evaluation refers to both IG and CG. There will be one separate questionnaire for the IG evaluating the different chapters of the intervention website in detail in order to collect data for the website’s improvement.

### Domains covered by the process evaluation

Following the MRC framework [30], the following domains will be covered by the process evaluation:

1. Implementation: fidelity, dose, reach (as no adaptations will be made to the study throughout the trial, the domain “Adaptations” is not covered)
2. Mechanisms of impact
3. Context.

#### Implementation: fidelity

Measuring to which extent the intervention is actually delivered as planned is difficult. There is still ongoing research on how to best collect data on fidelity [28]. Considering that the intervention is an online resource, the quality of the intervention will remain stable; therefore, mostly the received dose will vary. Fidelity could however be breached, if physicians talk about the meaning of the participant’s MRI throughout the study. Whether this is the case is assessed after the patient-physician encounter. Additionally, it is recorded if participants of the IG and CG really visited the respective website. Additionally, we will ask participants in the follow-up questionnaire (*t*_3_) and physicians after the patient-physician-encounter (*t*_2_) if they have been unblinded during the study.

#### Implementation: dose

The dose of an intervention can be subcategorized into:

- Dose *delivered*, i.e. the amount or number of intended units of each intervention or component delivered or provided by interventionists, and
- Dose *received*, i.e. the extent to which participants actively engage with, interact with, are receptive to and/or use materials or recommended resources; this can include “initial use” and “continued use”. The collection of data on the initial and continued use of the study materials, and information on what barriers and facilitators hinder or serve to maintain the implementation, can be used to interpret study outcomes.

In this trial, the dose *delivered* is the same for all participants, because the intervention is a website and therefore available at all times.

Concerning the dose *received*, the following aspects will be captured:

1. The use of the website (number of logins, duration of use, visited chapters)
2. Reasons for amount/lack of usage
3. Expenditure of the study.

#### Implementation: reach

The reach describes the proportion of the intended audience that participates in the intervention; it can be measured by attendance and includes documentation of barriers to participation. Demographic data is collected for all participants to properly describe the study population. In participants, reasons for participation as well as early exit will be registered (*t*_0_). PwMS who qualify for the study, but do not want to participate, will be asked for the main reason why (*t*_−1_).

#### Mechanism of impact

The domain *Mechanism of impact* covers the participants’ responses to, and interactions with, the intervention, mediators and unanticipated consequences. Overall, it examines how the intervention triggers change. For the evaluation, questionnaires with Likert scales, multiple choice and open questions will be used in IG and CG.

First, the satisfaction with the educational tool as well as relevance, importance of topics, understandability and handling of the website will be assessed. Participants of the IG will additionally answer a short questionnaire concerning the content of the intervention website. Secondly, barriers and promoting factors for the usage of the website (e.g. age, internet access and skills, amount of free time) will be assessed.

Thirdly, several of the primary and secondary outcomes (e.g. change in MRI-risk knowledge (subjective and objective) and attitude towards MRI) of the trial will be analysed to estimate how participants interacted with the website.

To monitor unintended consequences, threat by MS, change in quality of life, depression and anxiety or patient-physician-relationship/communication will be measured.

#### Context

Overall, 5 different MS centres are participating study centres; 3 of them are university hospitals, and 2 private practices. Depending on size and location, there is a variation in terms of the number of potential participants, practice hours, clinical focus of each clinic and access to an MRI. Therefore, there might be a difference in e.g. the number in prescribed MRIs or length of the waiting periods for patients.

As access to the internet may also vary in urban versus rural areas, internet availability and skills are assessed. Further, participants might also find information on MRI in MS on other platforms e.g. websites, blogs, magazines, books (…). Participants will therefore be asked, if they searched for additional information and what they found. Questions concerning contextual factors will be in the form of multiple choice and open questions (*t*_0_, *t*_1_, *t*_2_).

### Data analysis (process evaluation)

Data analysis of the process evaluation will follow descriptively (see above) and via SPSS (International Business Machines Corporation (IBM), Armonk, United States of America) or R (R Development Core Team) will be used. In case of a failed trial or inconclusive findings, it will be considered to use the quantitative data to determine questions for qualitative interviews.

### Summary process evaluation

The applied framework of Moore et al. [30] facilitates systematically appraising, analysing and retrieving relevant aspects of this complex intervention. The questionnaires are designed to permit an elaborate and more precise interpretation of the study results.

## Discussion

PwMS, while being constantly confronted with MRI results, possess a poor knowledge on the meaning of MRI in MS. They often do not feel competent to discuss their MRI results with their physician and receiving results may cause a relevant amount of fear [18]. At the same time, pwMS consider MRI to be very important and desire MRI education. The UMIMS trial is the first RCT to assess the effect of an MRI education in pwMS. It aims to prove that access to an education tool on MRI in MS will increase pwMS’ knowledge on this complex topic, facilitate communication with their physician and enhance SDM when discussing decisions involving MRI results. If the trial proves to be effective, the website is an easily accessible tool, which can be maintained and made available nationwide at a low cost.

### Trial status

The RCT is registered at clinicaltrials.gov (identifier NCT0387258). Recruitment has started in 15 March 2019 and is ongoing (estimated recruitment end 31 March 2021). Protocol version 2.0, date: 30 August 2020.

## Data Availability

The datasets used and/or analysed during the current study are available from the corresponding author on reasonable request.

## Abbreviations

CIS: Clinically isolated syndrome
CPS: Control Preference Scale
CR: Control group
DMD: Disease-modifying drug
EBM: Evidence-based medicine
EBPI: Evidence-based patient information
HADS: Hospital Anxiety Depression Scale
HAQUAMS: HAmburg QUAlity of life questionnaire in MS
IR: Intervention group
MAGNIMS: Magnetic Resonance Imaging in MS
MAPPIN’SDM: Multifocal Approach to Sharing in Shared Decision Making
MRC: Medical Research Council
MRI: Magnetic resonance imaging
MRI-EMA: MRI-emotions and attitude questionnaire
MRI-RIKNO: MRI-risk knowledge questionnaire
MS: Multiple sclerosis
NEDA: No evidence of disease activity
PDDS: Patient-determined disease steps
pwMS: People with multiple sclerosis
QoL: Quality of life
RCT: Randomized controlled trial
RRMS: Relapsing-remitting MS
SD: Standard deviation
SDM: Shared decision-making

## References

[1] Petersen G, Wittmann R, Arndt V, Gopffarth D (2014). Epidemiology of multiple sclerosis in Germany: regional differences and drug prescription in the claims data of the statutory health insurance. Nervenarzt..

[2] Thompson AJ, Banwell BL, Barkhof F, Carroll WM, Coetzee T, Comi G (2018). Diagnosis of multiple sclerosis: 2017 revisions of the McDonald criteria. Lancet Neurol.

[3] Kaunzner UW, Gauthier SA (2017). MRI in the assessment and monitoring of multiple sclerosis: an update on best practice. Ther Adv Neurol Disord.

[4] Río J, Ruiz-Peña JL (2016). Short-term suboptimal response criteria for predicting long-term non-response to first-line disease modifying therapies in multiple sclerosis: a systematic review and meta-analysis. J Neurol Sci.

[5] Filippi M, Rocca MA, Ciccarelli O, De Stefano N, Evangelou N, Kappos L (2016). MRI criteria for the diagnosis of multiple sclerosis: MAGNIMS consensus guidelines. Lancet Neurol.

[6] Fisniku LK, Brex PA, Altmann DR, Miszkiel KA, Benton CE, Lanyon R (2008). Disability and T2 MRI lesions: a 20-year follow-up of patients with relapse onset of multiple sclerosis. Brain..

[7] Chard D, Trip SA (2017). Resolving the clinico-radiological paradox in multiple sclerosis. F1000Res.

[8] Stangel M, Penner IK, Kallmann BA, Lukas C, Kieseier BC (2015). Towards the implementation of 'no evidence of disease activity’ in multiple sclerosis treatment the multiple sclerlosis. Ther Adv Neurol Disord.

[9] Heesen C, Kasper J, Fischer K, Kopke S, Rahn A, Backhus I (2015). Risk knowledge in relapsing multiple sclerosis (RIKNO 1.0)--development of an outcome instrument for educational interventions. PLoS One.

[10] Giordano A, Uccelli MM, Pucci E, Martinelli V, Borreani C, Lugaresi A (2010). The Multiple Sclerosis Knowledge Questionnaire: a self-administered instrument for recently diagnosed patients. Mult Scler.

[11] Brand J, Kopke S, Kasper J, Rahn A, Backhus I, Poettgen J (2014). Magnetic resonance imaging in multiple sclerosis—patients’ experiences, information interests and responses to an education programme. PLoS One.

[12] Zhang Q, Liao J, Liao X, Wu X, Wan M, Wang C (2014). Disease knowledge level is a noteworthy risk factor of anxiety and depression in patients with chronic obstructive pulmonary disease: a cross-sectional study. BMC Pulm Med.

[13] Bailey L (2010). Strategies for decreasing patient anxiety in the perioperative setting. AORN J.

[14] Sackett DL, Rosenberg WM, Gray JA, Haynes RB, Richardson WS (1996). Evidence based medicine: what it is and what it isn’t. BMJ..

[15] Bunge M, Muhlhauser I, Steckelberg A (2010). What constitutes evidence-based patient information? Overview of discussed criteria. Patient Educ Couns.

[16] Craig P, Dieppe P, Macintyre S, Michie S, Nazareth I, Petticrew M (2008). Developing and evaluating complex interventions: the new Medical Research Council guidance. BMJ..

[17] Cordier JF (2014). The expert patient: towards a novel definition. Eur Respir J.

[18] Engels K, Schiffmann I, Weierstall R, Rahn AC, Daubmann A, Pust G (2019). Emotions towards magnetic resonance imaging in people with multiple sclerosis. Acta Neurol Scand.

[19] Degner LF, Sloan Ja Fau-Venkatesh P, Venkatesh P. The control preferences scale. (0844–5621 (Print)). 9505581

[20] De Las Cuevas C, Penate W (2016). Validity of the Control Preferences Scale in patients with emotional disorders. Patient Prefer Adherence.

[21] Kasper J, Hoffmann F, Heesen C, Köpke S, Geiger F (2012). MAPPIN’SDM – the multifocal approach to sharing in shared decision making. PLoS ONE.

[22] Gold SM, Heesen C, Fau-Schulz H, Guder U, Monch A, Gbadamosi J, Buhmann C (2001). Disease specific quality of life instruments in multiple sclerosis: validation of the Hamburg Quality of Life Questionnaire in Multiple Sclerosis (HAQUAMS). Mult Scler.

[23] Zigmond AF, Snaith RP (1983). The hospital anxiety and depression scale. Acta Psychiatr Scand.

[24] Kopke S, Solari A, Rahn A, Khan F, Heesen C, Giordano A (2018). Information provision for people with multiple sclerosis. Cochrane Database Syst Rev.

[25] Altman DG (2009). Missing outcomes in randomized trials: addressing the dilemma. Open Med.

[26] Alshurafa M, Briel M, Akl EA, Haines T, Moayyedi P, Gentles SJ (2012). Inconsistent definitions for intention-to-treat in relation to missing outcome data: systematic review of the methods literature. PLoS One.

[27] Thabane L, Mbuagbaw L, Zhang S, Samaan Z, Marcucci M, Ye C, Thabane M (2013). A tutorial on sensitivity analyses in clinical trials: the what, why, when and how. BMC Med Res Methodol.

[28] Linnan L, Steckler A (2002). Process evaluation for public health interventions and research.

[29] Rahn AC, Kopke S, Kasper J, Vettorazzi E, Muhlhauser I, Heesen C (2015). Evaluator-blinded trial evaluating nurse-led immunotherapy DEcision Coaching In persons with relapsing-remitting Multiple Sclerosis (DECIMS) and accompanying process evaluation: study protocol for a cluster randomised controlled trial. Trials..

[30] Moore G, Audrey S, Barker M, Bond L, Bonell C, Hardeman W, et al. Process evaluation of complex interventions: a summary of Medical Research Council guidance. BMJ. 2015;350(1258):1–7. 10.1136/bmj.h1258 PMC4366184 25791983

